# Abscessed Teeth

**Published:** 1893-04

**Authors:** 


ARTICLE III.

### ABSCESSED TEETH.

Discusssion in the New York Odontological Society.

Dr. J. N. Farrar uses non-escharotic creosote in the treatment of pulpless teeth that are not associated with abscesses—wood creosote in combination with alcohol. The object of the alcohol is to enable the creosote to act in the presence of aqueous moisture, if there should be any in the tooth.

J. N. Farrar says: The plan of drying teeth with hot air is excellent, but I think bibulous paper will extract about as much water as the heating process, unless this is kept up for a long time. I do not regard moisture in the dentine as injurious to the tooth, but the pulp canal should be dry. If there are no abscesses, I fill the canal at the first sitting, if I have time. If I do not, I close it tightly. The great trouble with many dentists is that they fill temporarily with cotton saturated with some kind of drug, and then do not seal tight the canal. It should be sealed with something that will prevent any kind of micro-organisms from entering. I use phosphate of zinc cement over the cotton. When permanently filling a pulp canal I generally plug the apex with gold. What is needed is a small wad of indestructible substance.

Nature is our best guide in the treatment of an abscess. She makes a fistulous drainage from the socket. Drilling through the gums and socket, to the interior of the trouble, is the only scientific plan of treatment. I derived so much benefit from this treatment that in 1878 I gave a course of lectures on it at two colleges.

The ragged bone is generally necrosed bone. After an abscess has found vent through the side of the socket, the sac changes in its character. It hardens, in a measure disappears, and if the alveolar process behind the sac is deprived of its nutrient supplies it dies, and the sac dies in consequence, leaving a chamber filled with liquid substance. I call these cases sepulchral abscesses. The proper treatment, of course, is to go in there, and cut all the dead part out; cut with a sharp bur till the healthy tissue is reached; sometimes, however, it is difficult, if not impossible, to get all the dead bone out. In such instances there may be a temporary relief. Even if there is left some carious bone, it may, after a time, die—become necrosed.

Sometimes the end of the root projecting into this cavernous place itself takes on a degenerative condition. In fact, often the ends of these roots are rotten. These formerly plagued me much. Once I had a case that was favorable to amputate the root, and I did so. Of course that left the opening into the pulp chamber enlarged, so that it had to be filled with gold wire to make it tight; but it cured the abscess, and since that time the amputation of roots has, with me, been a common practice.

Concerning creosote as a remedy, I look on it in this way: If a sore is on the outside, an ulcer, we would not use creosote, nor would we use creosote in a flesh abscess just under the surface; we might perhaps wash it with something mild, such as castle soap and water, but with bone abscesses we are dealing with something different. Iodine preparations are disagreeable because they discolor, but they otherwise do very well. I think it is proper to cauterize the interior of a sac. Of course, there is no such thing as a "pyogenic membrane," but there is a sluggish surface which should be removed. When this is destroyed, and the parts are treated with some simple liquid, such as salt and water, injected every day or two till that degenerated substance is all driven out, and the contents are prevented from becoming rancid by continuing injections of salt and water, Labarraque's solution, or wood creosote and alcohol (which is non-escharotic) once in five or seven days, it will generally be followed by healthy granulations and a cure. For cauterization of a sac I have found escharotic creosote (coal tar creosote), or sulphate of zinc, as effective as anything. Either pump it through the canal of the tooth till it appears at the mouth of the fistula, or inject it through the fistula with a syringe.

Dr. Darby, of Philadelphia: Creosote and carbolic acid have been used in dentistry from time immemorial. Especially is this true of creosote, and, though it is not absolutely essential in a dental practice, it certainly has served, and is still serving, a very good purpose in the treatment of devitalized teeth and chronic alveolar abscess. We all know that many a sac at the apex of a root has been broken up, and a fistula healed by the escharotic effect of good creosote. The same may be said of carbolic acid. There may be better remedies than either, still I am not yet prepared to abandon good English creosots made by Squores, of London.

Hot air for drying the canals of teeth, to be effective, must have pressure behind it to force it into the root of the tooth. Some years ago I put into my laboratory an apparatus for giving me compressed air. It consists of the following:

A cylinder, made of galvanized iron, holding a hundred gallons, with a pipe running to my office, and at its end a gauge to denote the number of pounds pressure. A stop-cock within reach of the chair and a rubber tubing reaching to the mouth of the patient, with a Taft hot-air syringe, and a little spring cut off, to be held in the hand. In the laboratory, in close proximity to the cylinder, is an air-pump, and each morning the man pumps into this cylinder compressed air, from five to thirty pounds. By simply holding the bulb of my Taft hot-air syringe in the flame of my small Benson burner, I can have any degree of heat desired, and sufficient pressure back of it to force it into the root of the tooth.

To me this apparatus has been almost invaluable, as I am enabled to dry a tooth more thoroughly than I have ever been able to do in any other way.

When opening a devitalized tooth, in which I suspect septic conditions, my practice is to put into the pulp chamber a few crystals of the permanganate of potassium, and then washing it with its solution, after which I dry it with the hot-air blast, then wash it with absolute alcohol, and dry it this time with as much heat as the patient will endure.

Cotton has been recommended as a root filling, and in Philadelphia there are some who must believe in it, for they certainly use it a great deal. I have often removed cotton from the roots of teeth years after it had been put there, and the roots seemed in a healthy condition, and the cotton was not decomposed.

Drilling through the end of the root for relief in acute abscess is not often attended with pleasing result. My own experience and observation teaches me that the practice is a bad one, and leads to difficulty when it becomes desirable to find the root. But I do not believe in cutting or drilling through the alveolar process. This may do very well for the six anterior teeth, or for teeth with a single root, where the length can be ascertained, but when the abscess is at the apex of a double- or triple-rooted tooth the operation is too uncertain.

The first step is the thorough removal of the entire pulp *debris*, without which it is impossible to fill the canal properly. The opening up of the canal is best done in most cases with the flexible engine drill. There are comparatively few roots which cannot be opened up by this means to the very end; in the few which cannot, a fine flexible probe of piano wire, lubricated with a drop or two of carbolic acid, can be passed, with a little force, nearly, if not quite, to the apical foramen. The only exceptions to the rule to open the canal its entire length are those where the root is extremely crooked, rendering perforation likely, and those in which calcification has taken place. In the young the root canals are much larger than in older persons, and there is, therefore, greater necessity for opening the canal freely. Too many operators make the fatal mistake of not cutting away sufficient of the tooth to allow the drilling to be done on a line parallel to the direction of the root.

The next step is the perfect cleansing of the canal. Simply forcing the ground-up dentine out by means of an air or water syringe, and then swabbing with cotton pushed into the canal as far as the probe usually employed will carry it, will not answer. That is not perfect cleansing, and is far different from what I mean. Too few dentists, I am afraid, have the proper instruments for accomplishing what I wish understood by perfect cleanliness, which is a task requiring time and patience.

My method is, first, to prevent, as far as possible, the entrance of moisture into the canal during the drilling; then, having desiccated the powdered bone with a blast of hot, dry air, I work into the canal with a rotary movement a delicate, tapering probe, frequently withdrawing it to carry out the power adhering to its sides. This procedure is continued till the powder is completely removed. The hot blast is then again applied till the patient complains of discomfort from the heat, when Darby paper-points are used to ascertain if there is any moisture remaining at the extremity of the canal. Sometimes it will occur that serum or blood oozes from the vessels at the end of the root, and finds its way back into the canal, a fact which can be only ascertained by the measures just noted, unless the exudation is considerable. (When the apical foramen is large, or the root not fully developed, as in the teeth of young persons, it is difficult to check the extravasation of blood, requiring delicate surgical treatment to heal the wound before this filling of the canal should be attempted. When the filling is inserted, precaution must be taken against too much pressure on the freshly-healed surface, or the wound may reopen and complications follow.) With the tooth dried as far as the heat will penetrate, the remaining moisture in the canaliculi is absorbed, and the canal is ready for filling, unless the pulp is putrescent, the treatment in which case will be stated further on.

For filling root canals I have tried cotton, gold, amalgam, and cement, and long since discarded them, because, in my judgment, it was not possible to do perfect work with them, Not one of those mentioned can be made to enter and fill the canaliculi, which is an important part of the operation, as lessening the possibilities of the decomposition of the animal matter of the dentine. Nor can any one of them, in my judgment, be perfectly adapted to the walls, with the possible exception of cases where the roots are straight and short, and can be freely reamed out. I therefore use chlora-percha.

My method of introducing the chlora-percha is to flood the canal with chloroform, and, placing the patient in a position which will assist in the expulsion of air from the tooth, work the chloroform with a delicate probe to the end of the root. The thorough diffusion of the chloroform is, I believe, greatly assisted by capillary attraction, owing to the extreme dryness of the tooth. This is immediately followed by the introduction of gutta-percha canal points, which, by the aid of a to-and-fro motion, are dissolved in the chloroform along their entire length, forming the chlora-percha, which is distributed into every portion of the interior of the root accessible. Point after point is forced in, and placed in this manner, till the entire canal and the open canaliculi are filled with the chlora-percha.

I employ this method in all devitalized teeth, providing, of course, where the pulp is putrescent, for its deodorization and sterilization This, however, is a simple task; hot water and hot air are usually sufficient, few, if any, medicaments being required. When pericemental inflammation exists, I prefer the tincture of iodine to any other dressing, though some of the derivatives of this drug, as iodoform, aristol, etc., will accomplish perhaps equally good results by longer application. I see no necessity for the use of carbolic acid or creosote in the treatment of this condition, except to avoid a greater and more obnoxious odor.

I am opposed to treating alveolar abscess in any other way than by a positive and accurate procedure,—amputating the root, etc.

For years I relied on therapeutic measures, among them the injection of carbolic acid and creosote, though I employed the latter very rarely, partly because of its objectionable odor and partly because of my belief that carbolic acid was better. It was generally necessary to enlarge the apical foramen and force the medicine through, in the hope that its escharotic effect would destroy the sac and fistula, and thus the disease eventually disappear. Usually this method consumed much time, and only those that could well afford the luxury of such treatment received it. Great care is required in the use of carbolic acid lest it should accidentally come in contact with the gum. I have not infrequently observed much damage to the alveolus following its employment.

Early in my practice I realized the importance of positive surgical treatment in alveolar abscess, and for eight years I have employed that method almost exclusively, and, I am glad to say, with general success.

After the root has been cleansed and filled as before described, the next step is to complete the filling of the cavity in the tooth. This should be a permanent operation, as the tooth is never again to be opened. A local or general anæsthetic is now resorted to, as the patient or the case requires. If the fistula exists, the probe usually leads directly into the sac or the excavation in the alveolus, which is invariably present. If there is no fistula, or if it is at some distance from the seat of trouble, a direct opening into the sac must be made. For this purpose I prefer a spear-pointed drill which is thrust through the gum and alveolus in a direct line with the root. Practice and delicacy of touch shows where to drill and when to stop. A rose bur of suitable size is now inserted and rapidly revolved, to cut away the sac and necrosed bone. If the apex of the root is necrosed, it is likewise burred away, or if the necrosis is extensive, so much of the root is amputated as is necessary.

Amputation usually requires a larger opening through the wall of the alveolus, and in extreme cases a flap of the gum and alveolus is turned back to allow room for the saw and for the removal of the diseased tissue. Then cleanse the cavity and check the hemorrhage. Hot water injections will usually stop the bleeding, when an opportunity is afforded for ocular examination. Sterilize the wound, and if it is large, pack with antiseptic lint, continuing the dressing till no longer necessary. The antiseptic dressing may consist of injections of suitable solutions to aid in the healing of the parts.

If the operation has been inconsiderable, the wound may be left entirely alone after injecting with hot water and removing the *debris*, with the expectation of a cure. Even a fistula at some distance, necessitating a false opening into the sac, requires little treatment and soon disappears.

Generally the amputation can be accomplished at the time the root is filled, but to impress on you the importance of immediately curing all classes of abscesses with fistulous opening, it is only necessary to remind you that wherever found they are usually tubercular. If the antrum or nares is penetrated by a fistula, special treatment is commonly necessary.

I believe the root canal should never be left open, because so long as it remains open, resolution at the end of the root is practically impossible, owing to septic influence at the base of the newly-formed tissue, which again breaks down, unless the risk is provided against by immediate filling. To enlarge the apical foramen is complicating, by rendering more difficult the adaptation of a perfect filling, besides endangering the integrity of the new tissue by the likelihood of the filling material being forced beyond the root apex.

The operation of alveolotomy, as this process is called, is to my mind, based on a rational view of health and disease. An abscess is nature's way of getting rid of a disturbing influence, and often considerable damage to the surrounding parts and great suffering to the patient are the outcome of her efforts to dislodge the intruder. The indication is, therefore, to assist nature by removing the affected tissue which cannot be restored to health and usefulness. My vote is, therefore, for surgical procedure in both acute and chronic alveolar abscess,—some differences in the care of the two forms are required,—because experience has taught me that it is the best and quickest method to assist nature to assert herself.

Dr. Crouse, of Chicago: These surgical dentists, these men who get surgery chronic in their minds, think that the only way of doing anything is by a surgical operation. If there is any place I would steer my patients clear of, it is the office of one of these surgeons,—the one who thinks his work is to cut and slash all the time.

The trouble with surgical dentists is that they work at the wrong end. I have treated a great number of alveolar abscesses, and I have never found but three which did not get well with the treatment I will describe.

I happen to be in a field of colleges always productive of a great deal of surgery. We have five or six colleges in active operation. Do you not pity the patients when they have to submit to so many surgical operations a day? A law prohibiting dental surgeons or surgical dentists from operating, I think, would be a good thing. It might be a hardship to those who want to practice, but it would be a great blessing to the dear people.

What is the treatment I advocate? It is simple, especially simple since we have the rubber-dam, and can dry out the tooth and prevent the medicine from coming in contact with the tissues. What is the operation? Prepare your cavity; it is not necessary to give the details, except that care must be taken not to force the breach into the pulp canal, or get the cavity clogged with foreign matter; take a piece of soft India-rubber, cut it as near the size and shape of the cavity as you can; fill the cavity with carbolic acid, place the India-rubber into the cavity, and with it force the carbolic acid out through the fistulous opening. This is readily done with a blunt instrument and sudden force against the rubber, such force as is used in packing gold by hand-pressure. In my hands this has been the most effectual way of accomplishing the treatment. One such treatment is generally sufficient.

The frequent treatments described by the former speaker as being a luxury I am inclined to think my patients would not enjoy. I may be mistaken, and if I thought they wanted the pleasure of coming often and going through all that surgery, I do not know but I could work myself up to it.

Dr. Darby spoke of a class of cases where the pulp died under an oxychloride cap, and the root was found to be dry. In such cases I think the ends of the roots become encysted, and it is better to leave them alone than to treat them.

Dr. Van Woert: When you want to be certain, you must accept the inevitable, and fall back on this old reliable agent, carbolic acid.

We are all ready to hail with delight anything that may possibly do away with the use of this offensive drug, but as yet there has been nothing presented, to my knowledge, that can take its place. Carbolic acid has made a reputation for itself that I fear it will be hard to kill. My experience with the substitutes offered, so far, has been anything but satisfactory, and I am confident that Dr. Porter will regret it if he abandons its use.

I can see no reason for leaving the root canal open. After freeing it from the septic matter with which it is clogged, it is my practice to fill at once.

I am a strong advocate of immediate root-filling in all cases, for the following reasons: When a case of apical pericementitis presents itself, or any of the difficulties following, it is plain that such is the result of one of two causes—either mechanical injury or the presence of septic matter in the roots. If the latter, by removing the cause and restoring the parts to a thoroughly aseptic condition, nature, in ninety-nine out of a hundred cases, will take up the fight and set matters straight without the aid of surgery or medicaments.

The enlargement of the apical foramen I consider, as a rule, a bad practice, but there are cases where it is permissible and even advisable. The extraction and replanting of the tooth is at times really necessary; for instance, in excessive curvature or malformation of the roots.

I am a little surprised at his reasons for performing alveolotomy. I did not suppose there were any in this advanced age of surgery who would wait for the disease to produce a fistulous opening.

I fail to quite understand his reasons for surgical treatment in chronic abscess after the canal has been filled. If he means that no medicament whatever is to be used, I cannot agree with him. On the contrary, should he mean that by gaining access for evacuating the sac by surgical means, after which medicaments are used for the treatment of the case, I fully concur with him.

The following is what I use: Iodol; 10 grains; oxide zinc, 20 grains; oil cinnamon, 5 drops; carbolate of vaseline, a sufficient quantity to form a stiff paste at 140°.

This paste is so thick and dense that it can be rolled into shape very similar to the gutta-percha points manufactured for filling root canals. It is immaterial what the temperature of the surrounding parts may be, you cannot get inflammation enough to cause the least change of the vaselin, a difficulty which I had in the beginning from macerating the mixture cold.

Another useful remedy of great service in acute pericementitis is: One part tr. capsicum to two parts vin opii.

This, applied on absorbent cotton, a small piece of felt, or blotting paper, will relieve pain quicker than any local application I have ever tried.

Let me add one more word in favor of carbolic acid. In my opinion, sustained by a long experience, carbolic acid is the one of all drugs the results from which you can depend on. There has been a number of theories advanced for discarding it, one of which is the coagulation of albumen in the roots; and yet, notwithstanding this, the effect is satisfactory; and till we can prove all that is brought against it, we had best stand by it as an old friend.—*International.*

---

ARTICLE IV.

